# Stem cell therapy for diabetic foot ulcers: a review of preclinical and clinical research

**DOI:** 10.1186/s13287-018-0938-6

**Published:** 2018-07-11

**Authors:** Lara Lopes, Ocean Setia, Afsha Aurshina, Shirley Liu, Haidi Hu, Toshihiko Isaji, Haiyang Liu, Tun Wang, Shun Ono, Xiangjiang Guo, Bogdan Yatsula, Jianming Guo, Yongquan Gu, Tulio Navarro, Alan Dardik

**Affiliations:** 1 Vascular Biology and Therapeutics Program and Department of Surgery , Yale School of Medicine, Yale University, New Haven, CT USA; 2 Faculty of Medicine, Federal University of Minas Gerais, Belo Horizonte, Brazil; 3 Department of Vascular Surgery, Xuanwu Hospital, Capital Medical University, Beijing, China; 4 VA Connecticut Healthcare System, West Haven, CT USA

**Keywords:** Stem cell therapy, Cell therapy, Diabetic foot ulcer, Diabetic wound, Critical limb ischemia, Wound healing, Amputation

## Abstract

**Background:**

Diabetic foot ulcer (DFU) is a severe complication of diabetes, preceding most diabetes-related amputations. DFUs require over US$9 billion for yearly treatment and are now a global public health issue. DFU occurs in the setting of ischemia, infection, neuropathy, and metabolic disorders that result in poor wound healing and poor treatment options. Recently, stem cell therapy has emerged as a new interventional strategy to treat DFU and appears to be safe and effective in both preclinical and clinical trials. However, variability in the stem cell type and origin, route and protocol for administration, and concomitant use of angioplasty confound easy interpretation and generalization of the results.

**Methods:**

The PubMed, Google Scholar, and EMBASE databases were searched and 89 preclinical and clinical studies were selected for analysis.

**Results:**

There was divergence between preclinical and clinical studies regarding stem cell type, origin, and delivery techniques. There was heterogeneous preclinical and clinical study design and few randomized clinical trials. Granulocyte-colony stimulating factor was employed in some studies but with differing protocols. Concomitant performance of angioplasty with stem cell therapy showed increased efficiency compared to either therapy alone.

**Conclusions:**

Stem cell therapy is an effective treatment for diabetic foot ulcers and is currently used as an alternative to amputation for some patients without other options for revascularization. Concordance between preclinical and clinical studies may help design future randomized clinical trials.

## Background

The prevalence of diabetes mellitus has increased precipitously due to worldwide changes in nutrition and lifestyle, and is currently estimated to affect 425 million adults in 2017 and to increase to 629 million patients by 2045 [1]. Diabetic foot ulcer (DFU), a major complication of diabetes, is defined by The International Working Group on the Diabetic Foot as a full-thickness wound located below the ankle in a diabetic patient, and is associated with diabetic neuropathy and peripheral arterial disease [2]. More than 2% of the diabetic population develops a new foot ulcer each year leading to US$9.1 billion spent on care per year in the USA alone [3, 4]. In addition to pain, infection, amputation, and impaired mobility, DFUs are also associated with severe economic, social, and psychological sequelae. One amputation occurs every 30 s as a consequence of diabetic complications, and 84% of these amputations are preceded by a DFU [5, 6].

Current treatment guidelines for DFU recommend debridement, management of infection, revascularization, and off-loading pressure to promote healing [7]. However, ischemia, infection, neuropathy, and metabolic disorders frequently delay wound healing, a critical challenge for both patients and clinicians [8]. Recent advances in understanding the cellular and molecular complexities of wound healing have identified coagulation, inflammation, cell migration, and proliferation as critical steps required for tissue remodeling and healing [9]. Stem cell-based therapy has emerged as a promising therapeutic strategy to treat DFU. Stem cells synthesize and secrete cytokines that promote cell recruitment, immunomodulation, extracellular matrix remodeling, angiogenesis, and neuroregeneration, all of which promote wound healing [10–12]. Stem cells are also capable of differentiating into various cell types, such as myofibroblasts, keratinocytes, pericytes, and endothelial cells that may participate in wound healing [13].

Although initial reports of stem cell therapy have shown efficacy, the different parameters used within each of these studies prevents easy interpretation and generalization of these reports, and therefore recommendations for treatment of DFU with stem cell therapy are difficult to standardize [14]. This study reviews current literature reporting stem cell therapy for DFU, with specific attention to the type and origin of the stem cells used for treatment, routes of cell administration, use of granulocyte-colony stimulating factor (G-CSF), and adjunctive use and comparison to percutaneous transluminal angioplasty.

## Methods

**Fig. 1:**
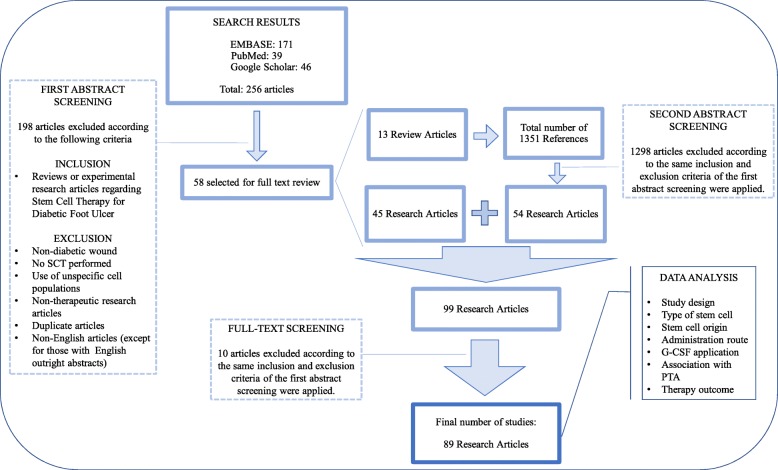
Diagram of study selection method and analysis. *G-CSF* granulocyte-colony stimulating factor; *SCT *stem cell therapy; *PTA *percutaneous transluminal angioplasty

The PubMed, EMBASE, and Google Scholar databases were searched on November 1, 2017. The search was performed using MeSH terms for “diabetic foot” paired with MeSH terms for “stem cell(s)” or “progenitor cells”, which resulted in 256 articles available for screening (Fig. 1). Abstracts for all these articles were screened, and review and experimental research articles describing stem cell therapy for diabetic foot ulcers were included. Articles were excluded if they were duplicated articles, nontherapeutic studies, nondiabetic wound trials, studies not employing stem cells, studies that used unspecified cell populations, and non-English articles with incomplete English abstracts.

Screening the initial 256 articles led to selection of 58 studies, consisting of 45 primary research studies and 13 reviews, which were reviewed in depth. A secondary screening was performed on the 1351 references obtained from the 13 reviews, yielding an additional 54 primary research studies, for a total of 99 primary research studies. Full-text review of these studies excluded an additional 10 articles, leading to the final inclusion of 89 primary research articles.

Each of the 89 research papers were examined in detail to determine study design (preclinical or clinical), stem cell type, stem cell origin, route of administration, use of G-CSF mobilization, and adjunctive use of percutaneous transluminal angioplasty.

## Results

### Study design

Of the 89 selected articles, there were 54 preclinical studies (60.7%) [15–68] and 36 clinical studies (40.4%) [38, 69–103]; one article reported data for both preclinical and clinical studies [38].

#### Clinical studies

One clinical study was retrospective [75] and 35 studies were prospective. Six studies were case reports [38, 85, 92–95] and 18 were case series [38, 69, 74, 77, 78, 80, 82–84, 86–90, 96, 99, 100, 102, 103]. Three were cohort studies [70, 76, 101], one was a case–control study [75], and eight were randomized clinical trials [71–73, 79, 81, 91, 97, 98]. The results for the eight randomized clinical trials selected among these studies are summarized in Table 1.

**Table 1:** Randomized clinical trials reporting stem cell therapy for diabetic foot ulcers

Author	Year	*N*	Study design	Type of cell	Administration route	Results	Follow-up (months)
Debin et al. [91]	2008	50	Two groups: - BM-MSC - Local wound treatment	Autologous BM-MSC	Intramuscular and subcutaneous	BM-MSC showed improved: - Rest pain (*P* < 0.01) - Claudication distance (*P* < 0.01) - Ulcer healing (*P* = 0.012) - Ankle-brachial index (*P* < 0.01) - Angiogenesis (*P* = 0.01) - Amputation rate (0.040)	3
Chen et al. [81]	2009	40	Two groups: - BM-MSC - Conventional individualized treatment	Autologous BM-MSC	Intramuscular	BM-MSC showed better: - Blood flow (*P* = 0.01)	3
Dash et al. [97]	2009	6**	Two groups: - BM-MSC - Local wound treatment	Autologous BM-MSC	Intramuscular	BM-MSC showed better: - Ulcer healing (*P* < 0.001)	3
Lu et al. [79]	2011	41	Two groups: - BM-MSC - BM-MNC	Autologous BM-MSC or BM-MNC	Intramuscular	BM-MSC showed better: - Ulcer healing (*P* = 0.022) - Limb perfusion (*P* = 0.040) - Ankle-brachial index (*P* = 0.017) - TcPO_2_ (*P* = 0.001) - Magnetic resonance angiography analysis (*P* = 0.018) No difference in pain relief and amputation rate	6
Jain et al. [98]	2011	48	Two groups: - BM-MSC - Peripheral blood	Autologous BM-MSC	Injection* and spray	BM-MSC showed better ulcer healing (*P* < 0.05)	3
Kirana et al. [73]	2012	24	Two groups: - BM-MSC - Tissue repair cells (TRC)	Autologous BM-MSC	Injection* and intraarterial	- BM-MSC 83% ulcer healing vs TRC 80% ulcer healing - BM-MSC and TRC had better TcPO_2_ (*P* = 0.092) - BMC-MSC improved ankle-brachial index (*P* < 0.10) - Angiogenesis detected in seven of the BM-MSC/TRC groups	12
Xu et al. [72]	2016	127	Eight groups: - Group A (G-CSF BID 5 μg/kg/day); four subgroups: 4, 5, 6 or 7 days - Group B (G-CSF BID 10 μg/kg/day); four subgroups: 4, 5, 6 or 7 days	Autologous PB-MSC	Injection* and topical*	G-CSF BID 5 μg/kg/day during 5 days is the optimal dose to mobilize EPC in DFU patients All groups reported improvement of life quality, pain, cold sensation, clinical symptoms and ulcer healing	1–15
Qin et al. [71]	2016	53	Two groups: - Angioplasty - Angioplasty and stem cell therapy	Allogeneic hUC-MSC	Intraarterial and intramuscular	Combination group showed better: - Ankle-brachial index (*P* < 0.05) - Skin temperature (*P* < 0.05) - Claudication distance (*P* < 0.05) - TcPO_2_ (*P* < 0.05)	1–3

^*^These studies did not specify the subtype of administration route. ^**^In this study, the n was 24 but only six patients h ad DFU; 18 patients were diagnosed with Buerger's disease. BID twice a day, *BM-MSC* bone marrow-derived mesenchymal stem cells, *BM-MNC* bone-marrow mononuclear cells, *DFU* diabetic foot ulcer, *EPC* endothelial progenitor cells, *G-CSF* granulocyte-colony stimulating factor, *hUC-MSC* human umbilical cord mesenchymal stem cells, *PB-MSC* peripheral blood-derived mesenchymal stem cells, *TcPO*_*2*_ transcutaneous oxygen pressure

#### Preclinical studies

The murine DFU model (31 articles) was most frequently used for preclinical research, with streptozotocin injections (30 articles) being the most common method to induce diabetes. Some of the most frequently observed parameters were a single wound model (22 articles), back wound location (30 articles), and wound diameter 5–6 mm (18 articles).

### Stem cell type

#### Adult stem cells

A total of 53 preclinical studies (98%) and all of the 36 clinical studies (100%) used adult stem cells for treatment (Table 2). Bone marrow-derived mesenchymal stem cells (BM-MSC) were the most frequently used cell type in both preclinical (*n* = 27; 50%) and clinical (*n* = 19; 53%) studies. Whereas adipose-derived stem cells (ADSC) were used in 11 preclinical studies (20%), only three clinical studies (8%) used this cell type. Human umbilical cord-derived mesenchymal stem cells (hUC-MSC) were used in 12 preclinical (22%) and four clinical (11%) studies. Two preclinical articles (4%) used peripheral blood-derived mesenchymal stem cells (PB-MSC), which was the second most frequent cell type in clinical studies (*n* = 11; 31%).

**Table 2:** Stem cell types advantages, disadvantages and use in clinical and preclinical studies

Stem cell type		Advantages	Disadvantages	Clinical studies		Preclinical studies	
Adult stem cells	BM-MSC	• Donor-specific therapy • Lower malignancy risk • Cell-lineage committed (targeting differentiation) • No ethical conflict	• Cell lineage committed (limited differentiation potential) • Biopsy high surgical risk • Nondisposable tissue • Low stem cell concentration • Cell concentration and performance influenced by comorbidities	19	(52.8%)	27	(50.0%)
	PB-MSC	• Donor-specific therapy • Lower malignancy risk • Cell-lineage committed (targeting differentiation) • No ethical conflict • Relatively disposable tissue • Vein puncture has low surgical risk • Simple cell harvesting protocol	• Cell lineage committed (limited differentiation potential) • Cell concentration and performance influenced by comorbidities • G-CSF administration needed	11	(30.5%)	2	(3.7%)
	hUC-MSC	• Future donor-specific therapy • Lower malignancy risk • Cell-lineage committed (targeting differentiation) • Disposable tissue • UC tissue harvesting has low surgical risk • Donor UCB banking storage	• Cell lineage committed (limited differentiation potential) • Immunoincompatibility • Ethical conflict • Low stem cell concentration • Need for UCB banking	4	(11.1%)	12	(22.2%)
	ADSC	• Donor-specific therapy • Lower malignancy risk • Cell-lineage committed (targeting differentiation) • No ethical conflict • Disposable tissue • Liposuction has low surgical risk	• Cell lineage committed (limited differentiation potential) • Cell concentration and performance influenced by comorbidities	3	(8.3%)	11	(20.4%)
Embryonic stem cells		• High differentiation potential (pluripotent)	• Increased malignancy risk • Ethical conflicts	0	(0.0%)	1	(1.9%)
Induced pluripotent stem cells		• High differentiation potential (pluripotent) • Somatic-cell memory (targeting differentiation) • Donor-specific therapy • No ethical conflict • Disposable tissue • Low cell harvesting procedure risk	• Increased malignancy risk • Complex induction protocol • Somatic-cell memory (biased differentiation)	0	(0.0%)	0	(0.0%)

*ADSC* adipose tissue-derived mesenchymal stem cells, *BM-MSC* bone marrow-derived mesenchymal stem cells, *G-CSF* granulocyte-colony stimulating factor, *hUC-MSC* human umbilical cord mesenchymal stem cells, *PB-MSC* peripheral blood-derived mesenchymal stem cells, *UC* umbilical cord, *UCB* umbilical cord blood

Although BM-MSC, PB-MSC, hUC-MSC, and ADSC were the most frequently used stem cell types, other stem cell types were used in some preclinical studies (Table 3). Kim et al. [60] reported enhanced wound healing with use of intradermal injections of human amniotic MSC in a murine DFU model, in comparison to human ADSC or human dermal fibroblasts. Similarly, Zheng et al. [18] related improved ulcer healing in diabetic mice with topical application of micronized amniotic membrane containing human amniotic epithelial cells compared to decellularized membrane. Lv et al. [16] demonstrated that human exfoliated deciduous tooth stem cells have similar healing potential as human BM-MSC in a rat diabetic model. Kong et al. [41] reported wound healing with intradermal injection of human placental MSC in diabetic Goto-Kakizaki rats. Badillo et al. [58] reported enhanced wound healing after injection of collagen gels containing embryonic fetal liver MSC in diabetic Lep db/db mice compared to CD45^+^ cell treatment. Barcelos et al. [29] used a collagen hydrogel scaffold to deliver human fetal aortic MSC in a murine DFU model.

**Table 3:** Studies reporting use of uncommon stem cell types

Author	Year	Species	Study design	Type of cell	Administration route	Results
Badillo et al. [58]	2007	Mouse	Three groups: - MSC - CD45^+^ - Control	Allogeneic, murine, embryonic, fetal liver MSC	Intradermal	MSC group showed smaller epithelial gap than CD45^+^ group (*P* < 0.004) MSC group showed increased granulation tissue area compared to control group (*P* < 0.05)
Barcelos et al. [29]	2009	Mouse	Three groups: - CD133^+^ cells - CD133^–^ cells - Control	Human fetal aorta-derived CD133^+^ progenitor cells	Collagen hydrogel	CD133^+^ group showed accelerated wound healing compared to control group (*P* < 0.05)
Lee et al. [53]	2011	Rat	Four groups: - Nondiabetic control rats - Diabetic rats treated with saline - Diabetic rats treated with saline and insulin - Diabetic rats treated with ESC and insulin	Mouse embryonic stem cells	Cell suspension drops	ESC and insulin-treated group wound healing accelerated compared to saline and insulin-treated group (*P* < 0.05)
Kim et al. [60]	2012	Mouse	Four groups: - Amniotic MSC - ADSC - Human dermal fibroblasts - Control	Human ADSC and human amniotic mesenchymal stem cells	Intradermal	Amniotic MSC group showed accelerated wound healing compared with ADSC, dermal fibroblasts or control groups (*P* < 0.01)
Kong et al. [41]	2013	Rat	Two groups: - Human placenta MSC - Control	Human placenta MSC	Intradermal	Placenta MSC group showed better wound closure compared to control group (*P* < 0.01)
Zheng et al. [18]	2017	Mouse	Three groups: - Living micronized amniotic membrane - Decellularized micronized amniotic membrane - Control	Human amniotic epithelial cells (HAECs)	Micronized amniotic membrane	Living membrane group had greater wound healing rate than decellularized membrane or control groups (*P* < 0.001)
Lv et al. [16]	2017	Rat	Three groups: - Exfoliated deciduous teeth stem cells - BM-MSC - Control	Human BM-MSC and human exfoliated deciduous teeth (SHED)	Local injection	SHED group showed accelerated wound healing compared to both BM-MSC and control groups (*P* < 0.05)

*ADSC* adipose tissue-derived mesenchymal stem cells, *BM-MSC* bone marrow-derived mesenchymal stem cells, *ESC* embryonic stem cells, *MSC* mesenchymal stem cells

#### Embryonic stem cells

One preclinical study (1.85%) and none of the clinical studies used embryonic stem cells (ESC; Table 2). Lee et al. [53] used topical mouse ESC in a rat DFU model; despite ESC xenotransplantation in immunocompetent rats, no rejection was observed and the use of pluripotent stem cells did not lead to tumor formation.

#### Induced pluripotent stem cells

The use of induced pluripotent stem cells **(**iPSC) for treatment of DFU has not been reported in any preclinical or clinical studies (Table 2). However, Gerami-Naini et al. [104] showed successful reprogramming of DFU-derived fibroblast cell lines into iPSC and further differentiation into fibroblasts. Okawa et al. [105] showed improvement of neural and vascular function in a polyneuropathy diabetic mouse model following transplantation of neural crest-like cells that were differentiated from murine iPSC. These findings suggest therapeutic potential for iPSC in the treatment of DFU.

### Granulocyte-colony stimulating factor

G-CSF is a cytokine that stimulates bone marrow to mobilize endothelial progenitor cells (EPC), increasing the number of available EPC for healing the DFU; G-CSF is found in wound tissue after acute injury [106]. In steady-state conditions, EPC typically circulate in low concentrations, and thus G-CSF is an important adjunct to promote increased yields of PB-MSC obtained for therapeutic purposes. G-CSF can also directly promote wound healing and reduce the number of surgical interventions in patients with a DFU [107, 108]. G-CSF was used in 10 clinical studies (Table 4); these studies used different protocols, with a dose range of 150–650 μg and a duration of G-CSF therapy varying from 3 to 6 days, prior to harvesting of BM-MSC and PB-MSC. Xu and Liang [72] found that injections of G-CSF 5 μg/kg/day for 5 days or 10 μg/kg/day for 4 days were the optimal G-SCF administration protocols to mobilize patients with a DFU receiving PB-MSC.

**Table 4:** Studies reporting use of Granulocyte-colony Stimulating Factor as part of stem cell therapy for diabetic foot ulcers

Author	Year	*N*	G-CSF dose	Duration (days)	Study design	Type of cell	Administration route	Results	Follow-up (months)
Kawamura et al. [87]	2005	21*	5 μg/kg/day	4	One group	Autologous PB-MSC	Intramuscular	Improvement of: - Rest pain** - Limb temperature** - Blood flow** 38% amputation	0.5–21
Yang et al. [100]	2005	34*	450–600 μg/day	5	One group	Autologous PB-MSC	Intramuscular	Improvement in: - Pain (87.1%)** - Cool feeling (90.3%)** - Ankle-brachial index (34.3%)** - TcPO_2_ (42.3%)** - Ulcer healing (40.0%)**	4
Kawamura et al. [99]	2006	59*	5 μg/kg/day	4	One group	Autologous PB-MSC	Intramuscular	Improvement in: - Symptoms (86%) - Skin temperature (53%) 89% amputation rate	9
Mao et al. [84]	2008	54*	500–600 μg/day	5	One group	Autologous PB-MSC	Intramuscular	Improvement in: - Limb pain (*P* < 0.05) - Coll feeling (*P* < 0.05) - Intermitted claudication (*P* < 0.05) - Ankle-brachial index (*P* < 0.05) - Skin temperature (*P* < 0.05) - Angiogenesis TcPO_2_ (42.3%) - Ulcer healing (40.0%)	6
Zhao et al. [83]	2009	15	500 μg/day	3	One group	Autologous BM-MSC	Intramuscular	13 patients showed improvement in: - Pain (*P* < 0.05) - Cold feeling (*P* < 0.05) - Angiogenesis (*P* < 0.01)	6
Zhou et al. [88].	2010	11	300 μg/day	3–4	One group	Autologous PB-MSC	Intraarterial	10 out of 11 patients had reduced pain, claudication, local cool-feeling and ulcer significant. Only one patient did not have any improvement	No details
Dubsky et al. [78]	2011	14	No details	No details	One group	Autologous BM-MSC and PB-MSC	Intramuscular	Patients showed improvement in: - TcPO_2_ (*P* = 0.0005) - Ulcer healing (*P* = 0.0078) - Pain (*P* = 0.002)	6
Dubsky et al. [76]	2013	50	5–8 μg/kg/day	6	Two groups: - Stem cell therapy - Control group	Autologous PB-MSC or BM-MNC	Intramuscular	Cell therapy group showed better: - Amputation rate (*P* = 0.009) - Ulcer healing (*P* = 0.0093)	6
Dubsky et al. [75]	2014	84	5–8 μg/kg/day	3–6	Three groups: - Stem cell therapy -Angioplasty - Control group	Autologous PB-MSC or BM-MNC	Intramuscular	PTA + SCT group showed better - TcPO_2_ (*P* < 0.05) - Amputation-free survival (*P* < 0.05) SCT group showed better - Wound healing in 3 months (*P* = 0.032), 6 months (*P* = 0.005), 12 months (*P* = 0.0013)	12
Tian et al. [70]	2016	61	150–300 mg/day	3–5	Three randomized groups: - PTA only - SCT only - PTA + SCT	Autologous PB-MSC	Intramuscular and intraarterial	PTA + SCT group showed: - Better total effective rate (*P* < 0.05) - Lower stenosis recurrence (*P* < 0.05)	9

^*^Number of DFU patients in studies that also included non-DFU patients. ^**^Results obtained from follow-up of DFU andnon-DFU patients. BM-MSC bone marrow-derived mesenchymal stem cells, *BM-MNC* bone-marrow mononuclear cells, *PB-MSC* peripheral blood-derived mesenchymal stem cells, *PTA *percutaneous transluminal angioplasty, *SCT *stem cell therapy, *TcPO*_*2*_ transcutaneous oxygen pressure

### Stem cell origin

Among the preclinical studies, only four (7%) examined autologous stem cell delivery; allogeneic stem cells were used in the majority of studies (*n* = 29; 54%).

Xenotransplantation was performed in 22 preclinical studies (41%) and all of them involved application of human stem cells in animal DFU models. In contrast, 32 (89%) of the clinical studies used autologous stem cells, and only four (11%) used allogeneic cells. No clinical studies used xenotransplantation to treat a DFU. These studies are summarized in Table 5.

**Table 5:** Stem cell origin advantages, disadvantages and use in clinical and preclinical studies

Stem cell origin	Advantages	Disadvantages	Clinical studies		Preclinical studies	
Autologous	• Immunoincompatibility • No ethical conflict • No infection transmission risk	• Lower stem cell concentration and limited healing potential • Cell harvesting procedural risk	32	(89%)	4	(7%)
Allogeneic	• Healthy stem cell source • No cell harvesting risk for DFU patient • Donor banking creation	• Relative immunoincompatibility • Need for disease screening • Ethical conflict	4	(11%)	29	(54%)
Xenotransplantation	• No ethical conflict • Healthy stem cell source • No cell harvesting risk for DFU patient • Donor baking creation	• High immunoincompatibility • Need for disease screening	0	(0%)	22	(41%)

*DFU* diabetic foot ulcer

### Administration route

#### Local administration

Nonvascular injections into tissue are currently the most commonly used route of administration to directly treat a DFU; injection was used in 28 preclinical studies (52%) and 31 clinical studies (86%) (Table 6). Intradermal (*n* = 11) and subcutaneous (*n* = 8) injections were more frequently used in the preclinical studies while the intramuscular (*n* = 24) route was more commonly used in clinical studies. Kwon et al. [57] reported increased wound strength in a rat DFU model treated with a single local injection of allogeneic BM-MSC; multiple intravenous injections did not significantly increase wound strength (*P* = 0.06), suggesting effectiveness of local injection.

**Table 6:** Stem cell therapy routes of administration routes; advantages, disadvantages and use in clinical and preclinical studies

Administration route		Preclinical studies		Clinical studies		Administration route subtype	Advantages	Disadvantages	Clinical studies		Preclinical studies	
Local	Injection	28	(52%)	31	(86%)	Intramuscular	• Simple • Low risk • Inexpensive	• High cell death • Low addressing and poor engraftment • No cell density and spacing control • May need debridement • Infection risk	24	(66.7%)	2	(3.7%)
						Subcutaneous and Intradermal			2	(5.6%)	19	(35.2%)
	Topical	23	(43%)	5	(14%)	Spray and Drops	• Painless • Simple • Low risk • Inexpensive	• High cell death • Low addressing and poor engraftment • No cell density and spacing control • May need debridement	3	(8.3%)	6	(11.1%)
						Hydrogel and Scaffold	• Low risk • Cell density and spacing control • Better retention and engraftment	•High protocol complexity • Expensive • May need debridement	0	(0.0%)	9	(16.7%)
Systemic	Endovascular	5	(9%)	6	(17%)	Intraarterial	• Can be performed during angioplasty • Possible immunomodulation and glucose homeostasis optimizing effect	• High surgical risk • Low addressing and poor engraftment • Expensive	6	(16.7%)	1	(1.9%)
						Intravenous			0	(0.0%)	4	(7.4%)

Topical administration was also frequently performed; topical delivery was used in 23 preclinical studies (43%) and five clinical studies (14%). Collagen hydrogels and scaffolds were the most commonly used vehicles to deliver cells [15, 17, 22, 29, 34, 58, 64]. Various other delivery methods were also used, including a silicon membrane with atelocollagen matrix to deliver murine ADSC [52], and artificial dermis containing human BM-MSC to treat two DFU patients [103]. Artificial dermis was also used for topical application of rat BM-MSC in a rat model [55], and to cover rat autologous ADSC sheets placed on a wound [20]. Micronized amniotic membranes have also been used [18]. Nanofibers containing human BM-MSC [32], human UC-MSC [33], or human ADSC [63] have also been used, as well as fibrin, both in gel to deliver human UC-MSC [54] and as a spray to deliver BM-MSC [38].

In preclinical studies, O’Loughlin et al. [34] and Falanga et al. [38] reported correlation between wound closure and the number of cells topically administered with collagen scaffolds and fibrin spray, respectively. In both studies, there was a significant difference in wound closure when at least 1 × 10^6^ cells were delivered.

#### Systemic administration

Endovascular stem cell delivery was performed in five preclinical (9%) and six clinical (17%) studies. Intraarterial femoral administration was performed in all six clinical studies while four preclinical studies used the intravenous tail vein route and only one study used the intraarterial femoral route. Zonta et al. [109] reported intraarterial stem cell therapy to be the most effective route for immunomodulatory purposes in rat kidney transplantation when compared to intravenous administration, reducing the incidence of tubulitis, arteritis, and glomerulitis (*p* < 0.01). Ho et al. [110] showed that multiple intravenous MSC doses positively impact glucose homeostasis in murine diabetic model, leading to a gradual decrease in blood sugar after two doses and total remission of diabetes within seven doses.

However, as a note of caution, a clinical study of 33 diabetic patients treated with autologous ADSC delivered endovascularly to treat critical limb ischemia reported formation of peripheral microthrombosis in two patients [111]; since diabetic ADSC released higher levels of plasminogen activator and lowered D-dimer formation, it was recommended to follow the D-dimer test prior to delivery of autologous ADSC to diabetic patients.

### Stem cell therapy and angioplasty

Angioplasty is currently an acceptable first-line treatment for selected patients with critical limb ischemia. In patients with critical limb ischemia contributing to the DFU, four clinical studies assessed the efficiency of percutaneous transluminal angioplasty that was performed in adjunctive fashion to the stem cell delivery, and one study compared both treatment options individually (Table 7). Tian [70] reported improved efficacy and reduced restenosis with combination treatment compared to either angioplasty or cell therapy alone. Similarly, intramuscular injection of human UC-MSC combined with angioplasty led to improved ankle-brachial index, claudication distance and transcutaneous oxygen pressure (TcPO_2_) compared to angioplasty alone [71, 101]. In a comparison of angioplasty and cell therapy, cell therapy was associated with superior wound healing despite similar TcPO_2_ and amputation-free survival [75]. Huang [90] reported enhanced wound healing after angioplasty in addition to intraarterial and intramuscular delivery of autologous PB-MSC.

**Table 7:** Studies reporting percutaneous transluminal angioplasty as part of stem cell therapy for diabetic foot ulcers

Author	Year	*N*	Study design	Type of cell	Administration route	Results	Follow-up (months)
Huang et al. [90]	2010	11	Prospective, one group: - PTA + SCT	Autologous PB-MSC	Intraarterial and intramuscular	Improvement of: • Ulcer healing 66.7% • Gangrene healing 77.8% • Pain 90.9% • Claudication distance 100% • Cold sensation 100% new vessels (*P* < 0.05)	3–12
Qin et al. [101]	2013	40	Prospective, NRS, two groups: - PTA only - PTA + SCT	Allogeneic hUC-MSC	Intraarterial	PTA + SCT group showed better: • ABI (*P* < 0.05) • Skin temperature (*P* < 0.05) • Claudication distance (*P* < 0.05) • Number of new vessels (*P* < 0.05)	3
Dubsky et al. [75]	2014	84	Retrospective, NRS, three groups: - PTA only - SCT only - Control (no intervention)	Autologous BM-MSC	Intramuscular	PTA + SCT group showed better: • TcPO_2_ (*P* < 0.05) • Amputation-free survival (*P* < 0.05) SCT group showed better • Wound healing in 3 months (*P* = 0.032), 6 months (*P* = 0.005), 12 months (P = 0.0013)	12
Qin et al. [71]	2016	53	Prospective, NRS, two groups: - PTA only - PTA + SCT	Allogeneic hUC-MSC	Intraarterial and intramuscular	PTA + SCT group showed better: • ABI (*P* < 0.05) • Skin temperature (*P* < 0.05) • Claudication distance (*P* < 0.05) • TcPO_2_ (*P* < 0.05)	1–3
Tian et al. [70]	2016	61	Prospective, RCT, three groups: - PTA only - SCT only - PTA + SCT	Autologous BM-MSC	Intraarterial and intramuscular	PTA + SCT group showed: • Better total effective rate (*P* < 0.05) • Lower stenosis recurrence (*P* < 0.05)	9

*NRS* non-randomized controlled study, *RCT* randomized clinical trial, *PTA* percutaneous transluminal angioplasty, *SCT* stem cell therapy, *BM-MSC* bone marrow-derived mesenchymal stem cells, *hUC-MSC* human umbilical cord mesenchymal stem cells, *PB-MSC* peripheral blood-derived mesenchymal stem cells, *TcPO*_*2*_ transcutaneous oxygen pressure, *ABI *ankle-brachial index

## Discussion

We report a comprehensive review of 89 preclinical and clinical investigations regarding the use of stem cells to treat DFU. We show that in both preclinical and clinical studies BM-MSC were the main cell type used, in over half the studies (Table 2), and cells were most commonly delivered by local injection (Table 6). As expected, autologous cells were used in the majority of clinical studies (89%) whereas preclinical studies frequently studied allogeneic and xenogeneic cells (Table 5). Cell number was rarely addressed; G-CSF was used in some studies prior to cell harvest of PB-MSC or BM-MSC, but without standardization of dose or protocol (Table 4). Stem cell therapy performed concomitantly with angioplasty showed more clinical effect compared to either of the therapies performed individually (Table 7).

Among all the studies of stem cell therapy for DFU, only eight of these studies are randomized clinical trials in human patients with DFU (Table 1). However, the heterogeneity among these trials prevents establishing strong conclusions, diminishing the power of any potential recommendations for clinical use of stem cell therapy to treat DFU. Thus, it is logical that future clinical trials should have comparable protocols, doses, cell types, and administration routes to allow good comparison of these expected studies. Unfortunately, the heterogeneity of the clinical trials is predictable from the heterogeneity of the preclinical studies, with differences in most of the parameters including wound models, types of stem cells, wound location, size, and control groups (Table 2).

The “best” stem cell type to treat DFU remains controversial. In both clinical and preclinical studies, predominant use of autologous adult stem cells (Table 2) is justified by simpler isolation protocols, safety, and absence of ethical conflict. While the clinical and preclinical studies commonly reported using bone marrow as the chief source for stem cells, the use of PB-MSC was much more frequent in clinical studies than preclinical studies. However, stem cell therapy with ADSC was far more prevalent in preclinical research, suggesting enthusiasm for using adipose tissue as a potential stem cell source. The fewer number of clinical studies using ADSC could be an artifact of the less convenient isolation process, with need to perform liposuction, as well as reports of an equivalent effectiveness of the stromal vascular fraction to treat DFU [19, 112] and also it was the most recent introduced adult stem cell type. Even though there are currently no reports regarding the use of iPSC to treat DFU, this novel cell source combines advantages of both adult and embryonic stem cells; future improvements in somatic cell induction techniques, as well as control of cell differentiation to prevent malignancy, may allow use of iPSC in the future.

Clinical studies mainly reported use of autologous cells, while allogeneic and xenogeneic cells were generally used for preclinical research. Autologous stem cell therapy poses minimal risk of infection, is immunocompatible, and is typically free of ethical or legal issues [113]. However, patients with DFU may have reduced autologous cell function due to the metabolic changes of diabetes as well as advanced age, thereby decreasing stem cell therapy effectiveness and increasing the risk of complications [111, 114–117]. The use of GCSF was observed to be advantageous in wound healing. Even though clinical studies differ regarding EPC mobilization protocol for PB-MSC stem cell therapy, 5 μg/kg injections BID for 5 days were reported as the optimal dose for DFU patients [72].

Alternatively, allogeneic therapy delivers stem cells from younger and healthier donors to the recipient but has the drawback of immunological incompatibility as well as potential legal issues; additionally, strict donor screening is needed to avoid disease transmission [113]. However, if these challenges are met, allogeneic stem cell therapy could be a good source of stem cells, allowing the formation of donation banks as well as potentially the use of cadaveric cells [118]. Immunological incompatibility is the major barrier to using xenogeneic cells. Interestingly, among preclinical studies reporting use of human cells in immunocompetent animal models, wound healing was observed without any immunological adverse effects. These results suggest the potential to use xenogeneic cells in the future.

Current evidence suggests that both local and systemic routes of stem cell therapy delivery are effective to heal DFU. Local injections of the cells were overall the most common method of cell delivery, with the distinction of intramuscular injections mostly being used for clinical studies, while preclinical studies predominantly used intradermal and subcutaneous injections (Table 6). Topical methods were frequently used in preclinical studies (43%), but less frequently in clinical studies (14%). Topical delivery within extracellular matrix scaffolds is another variable of interest. The extracellular matrix is a key modulator of cell maintenance, differentiation, proliferation, and self-renewal [119]; hydrogels and collagen scaffolds mimic the native in-vivo environment of stem cells, potentially increasing cell retention and engraftment [120–123] and even cell function [17].

Some commercially available bioengineered products and matrices are available. Graftjacket (Wright Medical Technology, Arlington, TN, USA) is an allogeneic skin graft obtained from donation banks that has demonstrated efficiency in wound treatment [124]. Bovine collagen scaffolds are available (Integra; Life Sciences Corp, Plainsboro, NJ, USA) and have been approved for burns and treatment of DFU [124]. Epifix (MiMedx, Marietta, GA, USA) is a dehydrated anionic membrane containing growth factors that is also a promising vehicle for stem cell therapy [124]. However, large-scale comparative effectiveness studies have not been performed.

## Conclusion

Current evidence points toward stem cell therapy as an effective treatment for human patients with DFU. Clinical and preclinical research studies do not offer a consensus regarding the optimal type of stem cell that should be used, and there is also no established optimal route or protocol to deliver stem cells. Differences within preclinical study designs suggest the need for a consensus regarding an optimal animal model that offers translation to human studies. Although autologous stem cells were the most commonly used stem cell type, it is possible that future studies will explore use of iPSC as well as allogeneic or even xenogenic cells. Administration of G-CSF promotes wound healing and its use is recommended as an adjunct to PB-MSC therapy. Hydrogels and bioscaffolds are promising topical delivery vehicles, but the impact of matrix design and configuration on stem cell function is still unknown. Angioplasty is a promising adjuvant to stem cell therapy in patients needing revascularization, and whether stem cell therapy will be used as an adjuvant to angioplasty remains to be determined.

## Abbreviations

ABI: Ankle-brachial index
ADSC: Adipose tissue-derived mesenchymal stem cells
BID: Twice a day
BM-MNC: Bone marrow-derived mononuclear cells
BM-MSC: Bone marrow-derived mesenchymal stem cells
DFU: Diabetic foot ulcer
EPC: Endothelial progenitor cells
ESC: Embryonic stem cells
G-CSF: Granulocyte-colony stimulating factor
hUC-MSC: Human umbilical cord mesenchymal stem cells
iPSC: Induced pluripotent stem cells
MSC: Mesenchymal stem cells
NRS: Non-randomized controlled study
PB-MSC: Peripheral blood-derived mesenchymal stem cells
PTA: Percutaneous transluminal angioplasty
RCT: Randomized clinical trial
SCT: Stem cell therapy
TcPO_2_: Transcutaneous oxygen pressure
UC-MSC: Umbilical cord-derived mesenchymal stem cells
